# Book Notices

**Published:** 1881-04-20

**Authors:** 


### BOOK NOTICES.

A MANUAL ON DISEASES OF THE EYE AND EAR, for the Use of Students and Practitioners, by W. F. Mittendorf, M. D., Surgeon to the New York Eye and Ear Infirmary, Ophthalmic Surgeon to Bellevue Hospital out-door department, assistant to the Chair of Ophthalmology and Otology, Bellevue Hospital Medical College. G. P. Putnam & Sons, New York, publishers, 1881.

This book, just received from its author, is a most admirable little treatise, adapted for the use of the student and practitioner, as its title indicates. The author's lectures to his private students, of which the book is made up, somewhat enlarged, were listened to with great interest and profit by the writer when a private pupil of his. Its pages are not cumbered with the treatment and surgical procedure of by-gone days, but contain only such as are in vogue at the present time. While it does not elaborately discuss the etiology and pathology of the diseases of the Eye and Ear, as much of it is given as the ordinary student of medicine or practitioner will care to study.

The painstaking manner in which it discusses treatment will recommend it as a hand-book of treatment to the general practitioner who is often called upon, especially in the country, to treat the minor diseases of the eye and ear. As a rule, the surgical methods are desirable, as they are now practiced in the New York Eye and Ear Infirmary.

This is the first treatise on the eye that describes Hotz' operation for trichiasis. The writer has often seen Dr. Mittendorf perform this little operation and with more satisfactory results than from any other he has ever witnessed for this most obstinate trouble of the upper lid.

About 70 pages are devoted to the Ear, and in the main the plan of St. John Roosa's admirable work is followed, but of course in an abridged form. The recognition of the ordinary diseases of the Eye will be facilitated by the fine illustrations taken from Sichel's Atlas, while diebreich and Wells are drawn on for some most excellent ophthalmoscopic plates of the retina. In addition to, these some plates from Politzen, representing the diseased conditions of the drum-head, will be found of great service. As the author well remarks: The importance of the study of the diseases of the Eye and Ear by every student of medicine is shown by the fact that many of the medical colleges, both of Europe and of this country have made the study of these diseases obligatory for graduation; he has, therefore, written this little book for the students' course, and for that purpose it is admirably adapted, because it is short and practical.

ARTHUR G. HOBBS, M. D. Atlanta, Ga.

A MANUAL OF DISEASES OF THE THROAT AND NOSE, By Frank Huntington Bosworth, A. M., M. D.

I have just finished reading this valuable addition to medical literature, and am so much pleased that I shall take the liberty of reviewing it In the special study of this branch of medicine I have never encountered a book that pleased me more. The style is clear, concise and to the point. While there is no want of modesty, still it is written with that decision that always comes with years of study, accompanied by large clinical experience. The author has shown the good judgment to be explicit and full when it is needed, and yet avoids all unnecessary discussion of conflicting views and opinions.

I have not the pleasure of Dr. Bosworth's acquaintance, yet from his book I should judge him to be a close student, a profound thinker, an accurate observer, and devoted to his specialty. I hope some day to have the opportunity to thank him for the pleasure and profit I have derived from his book. The chapters on instrumental examination are excellently written, and well illustrated. Those devoted to the anatomy, physiology and pathology of the mucus membranes fill a much needed want to the medical student who has not the time to cull these facts from the pages of Virchow and other eminent writers on histology and pathology. The section on "diseases of the fauces," contains much original thought. I think the suggestion of glycerine and iron in these affections to be very valuable.

In my opinion, however, by far the best chapters of the book are those on the "Diseases of the Nasal Cavities." unless I except the chapter on "Laryngeal Catarrh." Speaking of this affection the author says, (see page 276) "A large portion of cases are due to nasal catarrh. This may be overlooked while attention is directed entirely to the larynx, hence, both by physical examination and by question eliciting the subjective symptoms, a nasal catarrh, if present, should be discovered and removed by the proper remedies. The same may be said of pharyngeal catarrh, enlarged tonsils elongated uvula, etc. All remedies directed to the removal of the morbid condition within the larynx are of little avail so long as an exciting or aggravating cause exists to perpetuate the trouble." This is the secret of many failures in treating this affection, and is worthy of much thought and clinical investigation. The remainder of the book is well and forcibly written.

The last chapter is of interest as a history of the daring operation of extirpation of the larynx for the removal of growths malignant in character. At the close of the work is added an apendix giving the various formulæ that his experience, justifies him in recommending. In conclusion, I would recommend all who wish a complete and reliable treatise on this subject to add Dr. Bosworth's book to their library.

THOMAS F. HOUSTON, M. D., Atlanta, Ga.

THE CHEMISTRY OF MEDICINES—PRACTICAL. A Text Book for the use of Students and Pharmacists, embodying the Principles of Clinical Philosophy and their application to those Chemicals that are used in Medicine and in Pharmacy, including all those that are efficient in the Pharmacopœa of the United States, with fifty original cuts, by J. U. Lloyd, Prof. of Chimistry and Pharmacy of the city of New York, Associate Author of the Supplement to the American Dispensatory, Author of the Pharmacy and Chemistry of the Students' Pocket Medical Lexicon, Cincinnati. Published by the Author, 1881.

The author of the above work has sought to accomplish an important desideratum, the presentation, in a practical, plain and easily comprehended manner, of the medical student—giving "the theory more generally adopted by the majority of chemists of the day," avoiding unnecessary details and extremes. He remarks that "By introducing only medicinal chemicals I have been enabled to give, in most instances, quite thorough descriptions of the important ones. My laboratory and commercial experience have been drawn upon to render the work as complete and practical as possible, and my aim has likewise been to make it of interest and value to students, physicians and druggists."

The work is well gotten up in plain type, and contains 419 pages, with an ample index. It must prove a book of much practical interest and profit to the student, the physician and the druggist.

DIFFERENTIAL DIAGNOSIS: A manual of the comparative semiology of diseases, by De Howland Hall, M. D., Assistant Physician to the Westminster Hospital, London; Second American edition—extensive additions—Edited by Frank Woodbury, M. D., Physician to the German Hospital, Philadelphia. Philadelphia, D. S. Briton, 115 South Seventh Street, 1881.

This is a work of 218 large octavo pages, large plain type and excellent typographical execution. "*Hall's synopsis of the diseases of the lyrynx, lungs and heart*," is here enlarged to cover all the leading and important discoveries. Under the two heads of *General and Local*, the whole field is plainly and yet systematically considered. The work is practical and instructive, and will furnish a useful and important addition to the library of the intelligent and progressive physician.

HERNIA, STRANGULATED AND REDUCIBLE: with cure by subcutaneous injections, together with suggested and improved methods for relotomy; also an appendix giving a short account of various new surgical instruments, by Joseph H. Warren, M. D., member of American Medical Association, and delegate to foreign countries for 1880 and 1881; member of Massachusetts Medical Society, member Oswego County Medical Society, New York; member Boston Natural Historical Society; formerly Surgical and Medical Director United States, etc., with illustrations. Boston, Charles N. Thomas, 215 Tremont St., London. Sampson Low, Manston, Searle, and Revington, 1881—oc. pp., 28.

The difficult subject of Hernia is here treated in an able and lucid manner, and the new suggestions and new instruments presented will give to the work especial interest. The practitioner, and especially the surgeon, will find it a most useful and valuable addition to the medical library.

THE HEART AND ITS FUNCTIONS. New York, D. Appleton & Co., 1881. Price 50 cts.

This is one of the series of Health Primers prepared by eminent medical and scientific men of London. The present one is a work of 64 pages—very instructive and interesting.

